# Targeted Recovery of Phenolic Antioxidants from Grape Stems: A Sequential Approach

**DOI:** 10.3390/molecules30173546

**Published:** 2025-08-29

**Authors:** Violeta Jevtovic, Khulood Fahad Saud Alabbosh, Zoran Pržić, Jelena Nikolić, Reem Ali Alyami, Maha Raghyan Alshammari, Badriah Alshammari, Violeta Rakic, Odeh A. O. Alshammari, Milan Mitić

**Affiliations:** 1 Chemistry Department, College of Science, University of Ha’il, Ha’il 81451, Saudi Arabia; v.jevtovic@uoh.edu.sa (V.J.); reem-alyami1@hotmail.com (R.A.A.); maharaghyan@gmail.com (M.R.A.); badriah.alshammari@gmail.com (B.A.); odeh.alshammari@uoh.edu.sa (O.A.O.A.); 2 Biology Department, College of Science, University of Ha’il, Ha’il 81451, Saudi Arabia; k.alabosh@uoh.edu.sa; 3 Institute of Horticulture, Faculty of Agriculture, University of Belgrade, 11080 Belgrade, Serbia; zoranata4@yahoo.com; 4 Department of Chemistry, Faculty of Science and Mathematics, University of Niš, 18000 Niš, Serbia; jelena.cvetkovic@pmf.edu.rs; 5 Department of Agriculture and Food Technology Studies Prokuplje, Toplica Academy of Applied Studies, 18400 Prokuplje, Serbia; violetachem@gmail.com

**Keywords:** grape stems, *Vitis vinifera*, phenolic compounds, factorial design, extraction kinetics, thermodynamics, green extraction

## Abstract

Grape stems are an abundant by-product of winemaking and a promising source of phenolic antioxidants representing an underutilized biomass within the circular economy. Seven *Vitis vinifera* L. cultivars were analysed by HPLC DAD, with Merlot (Me), Cabernet Sauvignon (CS) and Italian Riesling (IR) identified as the richest sources. This comparative screening provided the basis for a multi-index optimization of extraction. A 2^3^ full factorial design (ethanol 30–60% *v/v*; 30–80 min; 25–65 °C) was used for optimization. The optimal green conditions—60% ethanol, 80 min, 65 °C—yielded 1.860 mg/g CA, 1.098 mg/g Q-gluc and 0.409 mg/g Q-glc, with the Merlot stems showing the highest extraction efficiency and Merlot consistently outperforming the other varieties. Kinetic modeling using an unsteady state diffusion model showed excellent agreement (R^2^ ≈ 0.99, RMS < 2%), suggesting a leaching-diffusion mechanism. The thermodynamic parameters confirmed an endothermic, spontaneous and irreversible process with ΔH° between 19.5 and 36.6 kJ/mol, ΔS° between 69.1 and 131.6 J/molK and ΔG° between −1.1 and −9.2 kJ/mol, depending on the compound and grape stem variety. This study shows that grape stems can be efficiently utilised as a sustainable source of phenolic antioxidants, with potential applications in the production of functional foods and dietary supplements. This integration highlights the novelty of the study and supports the valorization of grape stems in the framework of sustainability and the circular economy.

## 1. Introduction

The wine industry plays an important role in the European economy, culture and heritage and has made this region one of the most important wine producers in the world. However, wine production generates significant agro-industrial by-products, including grape pomace, seeds, berry skins, vine pruning wood and grape stems [1,2,3,4]. If not managed properly, these residues can lead to environmental problems such as soil degradation, water pollution and greenhouse gas emissions [5]. A more sustainable management of these residues is essential, and their valorization aligns with circular economy principles in the wine industry.

Grape stems are removed during grape processing, before fermentation (vinification) to avoid excessive astringency and to preserve the desired typical varieties sensory characteristics of the wine [6,7]. Depending on varieties, grape stems generally account for between 1.4% and 7.0% of the total grape mass processed during winemaking [8]. Despite their large quantity, grape stems currently have little commercial value and are mostly used as livestock feed or for soil enrichment. However, recent studies suggest that grape stems may represent an underutilized source of dietary fiber and bioactive compounds, especially antioxidants [9,10]. Among these, phenolic compounds stand out due to their well-documented health-promoting effects. The phenolic profile of grape stems contains remarkable amounts of flavan-3-ols, hydroxycinnamic acids, monomeric and oligomeric flavonols and stilbenes [11,12]. Recent research has also demonstrated their richness in flavonoids such as catechins, proanthocyanidins (including B1–B3 dimers), quercetin and various glycosylated derivatives [12,13]. Nevertheless, grape stems are increasingly recognized as an underutilized resource rich in phenolic compounds with potential high-value applications.

Catechins, important components of grape stems, are known for their strong antioxidant capacity and remarkable physiological effects [14,15]. Recent studies have shown that their antioxidant activity is highly dependent on the number of hydroxyl groups and the presence of certain structural features within the molecule. These compounds are particularly effective in scavenging reactive oxygen and nitrogen species, which contributes to their role in mitigating oxidative stress [16].

Given their multiple health-promoting effects, the inclusion of catechin rich products in the daily diet is increasingly encouraged [17]. Among the various biological activities attributed to catechins, their antioxidants, anti-inflammatory and chemopreventive properties are considered the most important [17,18].

Grape stems have been identified as a particularly rich source of catechins. In a comprehensive analysis of stem extracts of different grape varieties, Leal et al. (2020) [19] reported catechin (a flavan-3-ol) as the predominant phenolic compound. Similarly, Serra et al. (2023) [4] quantified (+)-catechin concentrations ranging from 0.120 to 1.858 mg/gram of grape stem. It is noteworthy that the (+)-catechin concentrations in the grape stems are higher than in the skins and seeds of the grapes. This compound is very sensitive to oxidative degradation, but under such conditions it exerts a protective function by neutralizing free radicals and protecting cellular macromolecules [20]. Despite these advances, most studies have focused on single varieties or limited extraction parameters, leaving gaps in comparative and optimization approaches.

In addition to catechins, quercetin and its derivatives are also abundant in grape stems and exhibit remarkable biological activity. As a flavonol, quercetin is considered one of the most powerful natural antioxidants, its effectiveness being closely linked to the number of hydroxyl groups in its structure [21]. The aglycone form of quercetin can scavenge free radicals and chelate transition metal ions, thereby mitigating oxidative stress, which is a major contributor to various degenerative diseases [22,23]. In addition to its antioxidant function, quercetin also has antibacterial and therapeutic properties [24]. These compounds are already of considerable industrial interest, particularly in the nutraceutical and functional food sectors, where catechins and quercetin derivatives are widely used as natural antioxidants. At present, they are mainly sourced from tea, onions, and other plant matrices, but yields are often low and extraction costs high. In this context, grape stems represent a promising alternative raw material, as their recovery not only provides competitive yields but also contributes to sustainable by-product valorization.

The aim of this study was to optimize the maceration extraction of three health-relevant phenolic compounds—quercetin-3-glucuronide (Q-gluc), quercetin-3-glucoside (Q-glc) and (+)-catechin (CA) from grape stems from seven different grape varieties. With the development of food science, the investigated bioactive molecules are becoming increasingly important, particularly due to their pronounced antioxidant potential and their specific nutritional and properties. These bioactive molecules with strong antioxidant potential are considered promising ingredients for the development of functional foods, dietary supplements and natural health products. Although phenolic compounds are widely available in plant sources, their extraction from agro-industrial by-products is particularly attractive due to the added value they bring to waste streams and their importance for sustainable production [25,26]. This study addresses these gaps by integrating variety-specific comparisons with multi-index factorial optimization, combined with kinetic and thermodynamic modeling, to provide deeper mechanistic and applied insights.

In the present study, a stepwise approach was followed. First, seven grape stem varieties were analysed for their phenolic profiles. Based on the results, three varieties with the highest content—‘Merlot’, ‘Cabernet Sauvignon’ and ‘Italian Riesling’—were selected for extraction optimization using a 2^3^ full factorial design. The effects of ethanol concentration, extraction time and temperature on phenolic content were systematically evaluated. Kinetic and thermodynamic modelling was applied to better understand the dynamics of the extraction. Finally, the extracts obtained under optimal conditions were analysed for total phenolic and flavonoid content and antioxidant activity. This integrated strategy provides a comprehensive framework for the valorization of grape stems as a sustainable and efficient source of natural antioxidants and provides additional insights for further research. This aligns with sustainable winemaking practices and supports the valorization of grape by-products within circular economy frameworks.

## 2. Results and Discussion

Grape stems as second product in the wine industry are an inexpensive and rich source of natural antioxidants with proven health-promoting potential, making them valuable for use in dietary supplements and phytochemical applications. Our screening results identified Italian Riesling, Cabernet Sauvignon, and Merlot stems as the most promising cultivars, with combined contents of (+)-catechin (CA), quercetin-3-glucuronide (Q-gluc), and quercetin-3-glucoside (Q-glc) accounting for 59%, 68%, and 78% of total phenolics, respectively [13]. To enable their use in food and pharmaceutical formulations, further screening and targeted optimization of the extraction process using safe, non-toxic solvents is essential for maximizing recovery of these valuable compounds.

### 2.1. Individual Phenolic Composition of Grape Stems

As part of the initial screening, the phenolic profiles of seven grape stem varieties were analyzed to quantify the content of key bioactive compounds (as described in Section 2.2). The concentrations of individual phenolic compounds are presented in Table 1. The total identified phenolic content, calculated as the sum of all quantified compounds, ranged from 1.465 to 4.783 mg/g of dry matter. Among the identified compounds, (+)-catechin (CA) was the most abundant, followed by quercetin-3-glucuronide (Q-gluc).

The highest levels of CA, Q-gluc, Caf, and Cm were observed in Merlot (Me), Cabernet Sauvignon (Cs), and Italian Riesling (IR). Merlot stems also showed the highest content of Q-glc, while L-glc was most concentrated in both Merlot and Italian Riesling samples.

When considering the sum of the three target compounds—CA, Q-gluc, and Q-glc—these accounted for 79.15% of the total phenolics in Merlot, 74.88% in Cabernet Sauvignon, and 70.44% in Italian Riesling. Based on this predominance, these three phenolic compounds were selected as the focus for optimization, and the corresponding grape stem varieties (Me, Cs, IR) were chosen for further study.

The extraction process was optimized using a 2^3^ full factorial design, with ethanol concentration (x_1_), extraction time (x_2_), and extraction temperature (x_3_) as independent variables. The dependent variables were the yields of Q-gluc, Q-glc, and CA in the selected grape stem samples. Process levels for each factor were selected based on preliminary screening experiments that indicated their influence on phenolic recovery.

### 2.2. Quercetin-3-Glucuronide (Q-Gluc)

The experimentally determined concentrations of Q-gluc under various extraction conditions are presented in Table 2. Values ranged from 0.217 to 0.984 mg/g, depending on the grape stem variety and extraction parameters. The highest Q-gluc yield was obtained from Merlot stems using 60% ethanol at 65 °C for 80 min. In contrast, the lowest yield was recorded for Italian Riesling stems under conditions of 30% ethanol, 25 °C, and 30 min of extraction.

The ANOVA results are summarized in Table 3. The significance of the main factors, as well as their two-way and three-way interactions, was evaluated based on the corresponding F- and *p*-values. Higher F-values indicate a stronger effect on the model, while *p*-values less than 0.05 denote statistical significance.

To simplify the regression model, all terms with *p*-values ≥ 0.05 were considered statistically insignificant and excluded. The resulting reduced regression equations are as follows:

(1) $$y_{IR}=0.3201+0.0389x_{1}+0.0266x_{2}+0.0421x_{3}+0.0064x_{1}x_{2}+0.0039x_{1}x_{2}x_{3}$$

(2) $$y_{CS}=0.3347+0.0472x_{1}+0.0387x_{2}+0.0440x_{3}+0.0053x_{1}x_{2}+0.0060x_{1}x_{3}+0.0055x_{2}x_{3}$$

(3) $$y_{Me}=0.6696+0.0991x_{1}+0.0856x_{2}+0.0899x_{3}+0.0131x_{1}x_{2}+0.0129x_{1}x_{3}+0.0119x_{2}x_{3}$$

The predicted values generated by the polynomial regression model showed strong agreement with the experimentally observed data (Table 2), confirming the model’s accuracy in predicting Q-gluc yields from grape stems. This high level of agreement demonstrates that the developed regression model is well-suited for describing the extraction process.

The coefficient of determination (R^2^) exceeded 99%, indicating excellent model fit, while adjusted R^2^ values remained above 98% for all responses. Additionally, the coefficient of variation (CV) was below 2%, further supporting the model’s precision, reliability, and robustness.

ANOVA results revealed that all three linear factors—ethanol concentration (x_1_), extraction time (x_2_), and temperature (x_3_)—were statistically significant, with F-values ranging from 257.77 to 645.27. These findings confirm their strong influence on Q-gluc content.

**Figure 1:**
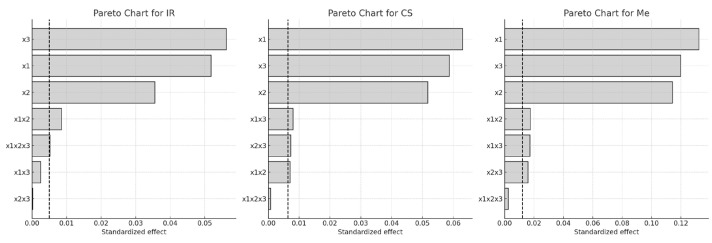
Pareto charts of factors influence on Q-gluc content in extracts of different grape stems: x_1_, ethanol concentration; x_2_, extraction time; x_3_, extraction temperature (Dashed line indicates the significance limit for standardized effects).

The Pareto chart (Figure 1) further illustrates the relative impact of each factor. The length of each horizontal bar represents the magnitude of the standardized effect, while the dashed line marks the 95% confidence threshold for statistical significance.

Among the factors examined, extraction temperature (x_3_) had the most pronounced impact on Q-gluc yield, followed by ethanol concentration (x_1_), particularly in the IR and CS samples. For Merlot (Me), the order of influence was x_1_ > x_3_ > x_2_. All two-way interactions were found to significantly affect Q-gluc extraction in CS and Me, while the three-way interaction (x_1_·x_2_·x_3_) showed no statistical relevance. In contrast, this three-way interaction was statistically significant in the case of IR (*p* < 0.05).

**Figure 2:**
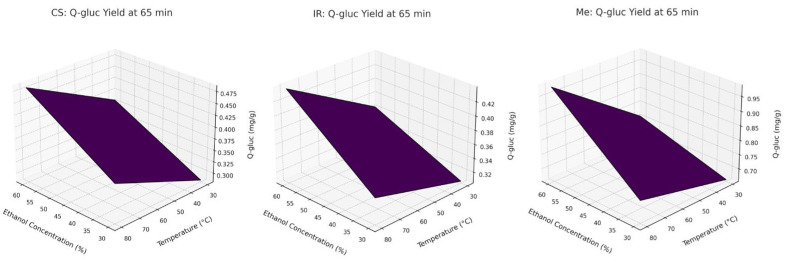
Response-surface plots for Q-gluc extraction.

To further visualize these interaction effects, response surface plots are presented in Figure 2. These 3D surfaces clearly demonstrate that temperature and ethanol concentration have the strongest combined influence on Q-gluc extraction, whereas extraction time has a relatively minor effect within the tested range. The plots were derived from the full factorial design and align with the trends observed experimentally for Q-gluc.

**Figure 3:**
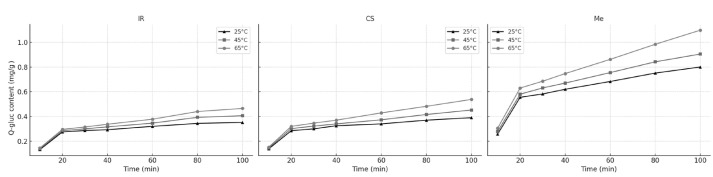
Variation in Q-gluc contents in extracts during the extraction process at (▲) 25 °C; (▪) 45 °C; and (•) 65 °C; for Ir, CS, and Me grape stems.

In the next phase of the study, the kinetic and thermodynamic parameters of Q-gluc extraction were evaluated for stem samples from three grape varieties. Figure 3 presents the variation in Q-gluc concentration over time for Italian Riesling (IR), Cabernet Sauvignon (CS), and Merlot (Me) stems, extracted at different temperatures.

During the initial extraction phase, which lasts approximately 20 min, the process is primarily governed by a leaching mechanism. In this stage—often referred to as the fast extraction phase—the solvent quickly dissolves Q-gluc that is readily accessible on the surface of the plant particles, resulting in a rapid increase in extract concentration over time.

Following this initial period, the extraction rate declines markedly as the process becomes controlled by internal diffusion. In this slower phase, Q-gluc diffuses from the interior of the plant matrix into the solvent, leading to a more gradual rise in concentration. This phase is known as the slow extraction or diffusion phase and is significantly slower than surface leaching.

To describe the observed extraction kinetics of Q-gluc, the experimental data were fitted to an unsteady-state diffusion model (Equation (6)). Kinetic parameters were estimated through linear regression based on the linearized form of the model equation.

The results revealed that temperature plays a key role in the slow extraction phase. An increase in extraction temperature led to a higher diffusion rate constant (k), indicating enhanced mass transfer from the plant matrix. The model’s fit quality was assessed using the coefficient of determination (R^2^) and the root mean square (RMS) error. As shown in Table 4, all R^2^ values exceeded 0.80, and RMS values remained below ±10%, confirming that the model adequately describes the experimental data across all grape stem varieties.

Figure 3 and Table 4 illustrate how Q-gluc concentrations change over time for different grape stem types. These variations resulted in distinct leaching and diffusion rate constants, reflecting the diverse physical and chemical characteristics of raw materials. Differences in cell wall thickness, pore structure, and initial phenolic content are likely contributors to the observed variation in extraction behavior.

The activation energy (Ea) for Q-gluc extraction from grape stems ranged between 8.47 and 10.96 kJ/mol, which aligns well with previously reported values. For comparison, Bucić-Kojić et al. (2007) [27] reported activation energies between 1.10 and 7.70 kJ/mol for polyphenol extraction from grape seeds using 50% aqueous ethanol. In general, lower activation energy indicates a faster extraction rate, as more molecules possess sufficient energy to overcome the energy barrier under given conditions.

The positive values of enthalpy (ΔH°) and entropy (ΔS°) across all tested temperatures confirmed that Q-gluc extraction using 60% ethanol is an endothermic and irreversible process. The Gibbs free energy change (ΔG°) ranged from −1.12 to −4.88 kJ/mol, indicating that the extraction was spontaneous and thermodynamically feasible under all conditions. Moreover, the increasing negativity of ΔG° with temperature suggests that spontaneity improves at higher extraction temperatures.

Among the tested conditions, extraction from Merlot (Me) stems at 65 °C proved most favorable, characterized by the lowest activation energy, the highest diffusion and equilibrium constants (k and K), and the most negative ΔG°, confirming both efficiency and spontaneity of the process.

### 2.3. Quercetin-Glucoside (Q-Glc)

The experimental results obtained for Q-glc during the optimization process are presented in Table 5. The highest Q-glc yield (0.386 mg/g) was achieved using 60% ethanol at 65 °C for 80 min in Merlot (Me) grape stems. Conversely, the lowest yield (0.045 mg/g) was recorded under the mildest conditions—30% ethanol, 25 °C, and 30 min—in Italian Riesling (IR) stems.

The statistical evaluation of the extraction process using ANOVA is summarized in Table 6.

The resulting simplified regression equations are:

(4) $$y_{IR}=0.0659+0.0084x_{1}+0.0066x_{2}+0.0086x_{3}$$

(5) $$y_{CS}=0.0977+0.120x_{1}+0.0107x_{2}+0.0135x_{3}$$

(6) $$y_{Me}=0.2677+0.0292x_{1}+0.0320x_{2}+0.0420x_{3}+0.0055x_{1}x_{3}+0.0057x_{2}x_{3}$$

The predicted values of Q-glc content, calculated using regression Equations (4)–(6), are presented in Table 5. A comparison between predicted and experimental values shows strong agreement, confirming that the polynomial regression models accurately describe Q-glc extraction behavior under the tested conditions.

This strong correlation is further supported by the high coefficient of determination (R^2^), which exceeded 0.96 for all models (Table 6), indicating that over 96% of the variability in Q-glc yield can be explained by the selected factors.

For Italian Riesling (IR) and Cabernet Sauvignon (CS), extraction temperature (x_3_) had the greatest influence on Q-glc content, followed by ethanol concentration (x_1_) and extraction time (x_2_). None of the two- or three-way interactions were statistically significant for these two varieties. In contrast, for Merlot (Me), the factor influence followed the order x_3_ > x_2_ > x_1_, with two significant two-factor interactions: x_1_·x_3_ and x_2_·x_3_.

**Figure 4:**
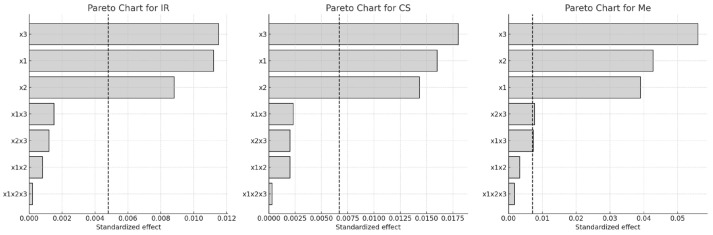
Pareto charts of factors influence on Q-glc content in extracts of different grape stems: x_1_, ethanol concentration; x_2_, extraction time; x_3_, extraction temperature (Dashed line indicates the significance limit for standardized effects).

**Figure 5:**
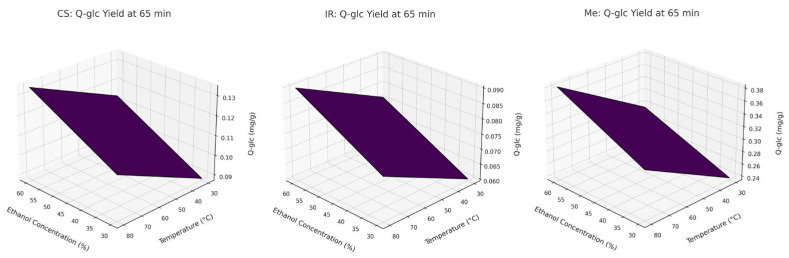
Response-surface plots for Q-glc extraction.

These findings are further illustrated in Figure 4 (Pareto chart) and Figure 5 (3D response surface plots), which visualize the relative importance and combined effects of the extraction parameters on Q-glc yield.

**Figure 6:**
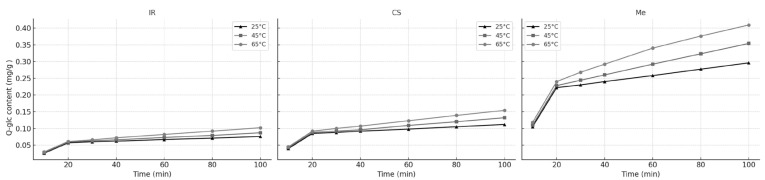
Variation in Q-glc contents in extracts during the extraction process at (▲) 25 °C; (▪) 45 °C; and (•) 65 °C; for Ir, CS, and Me grape stems.

The effect of different extraction times (10, 20, 30, 40, 60, 80, and 100 min) on Q-glc extraction from different stems are presented in Figure 6. All extraction was carried out at 25, 45, and 65 °C with 60% ethanol.

As with Q-gluc, the extraction of Q-glc followed a two-phase pattern: an initial washing phase during the first ~20 min, followed by a slower diffusion-controlled phase. The kinetic parameters calculated using the unsteady-state diffusion model at different temperatures are presented in Table 7.

Activation energy (Ea) is a key parameter for understanding the dynamics of the extraction process [28]. Its value can be influenced by several factors, including the structural properties of the plant matrix and the target compound, pre-treatment of the sample, and the nature of the extraction solvent [29].

The activation energies (Ea) calculated for the slow extraction phase using the unsteady-state diffusion model are presented in Table 7. The values were 12.49 kJ/mol for Italian Riesling (IR), 11.39 kJ/mol for Cabernet Sauvignon (CS), and 12.68 kJ/mol for Merlot (Me). These results suggest that Q-glc extraction from Merlot stems was more temperature-dependent, while the extraction from CS was less affected by temperature changes. Although specific Ea values for Q-glc extraction from grape stems have not been previously reported, the obtained results are consistent with values observed for other phenolic compounds in plant matrices.

Thermodynamic analysis further supports these findings. The extraction process was characterized by positive entropy changes (ΔS°: 84.47–129.18 J/mol·K) and positive enthalpy values (ΔH°: 26.53–36.58 kJ/mol), confirming the endothermic and irreversible nature of the process. The negative Gibbs free energy values (ΔG°: –1.40 to –7.08 kJ/mol) indicate that the extraction is spontaneous under all tested conditions (Table 7). Among the tested samples, the most favorable conditions for Q-glc extraction were observed for Cabernet Sauvignon at 65 °C, which exhibited the lowest Ea, highest kinetic and equilibrium constants (k and K), and the most negative ΔG°, indicating a more efficient and spontaneous process.

### 2.4. Catechin (CA)

The results obtained using a 2^3^ experimental design for CA extractions from the stems of three different grape varieties are shown in Table 8. The ANOVA results are presented in Table 9.

The resulting simplified regression equations are:

(7) $$y_{IR}=0.7087+0.0645x_{1}+0.0870x_{2}+0.0810x_{3}+0.0087x_{1}x_{2}+0.0067x_{1}x_{3}+0.0102x_{2}x_{3}$$

(8) $$y_{CS}=0.6764+0.0586x_{1}+0.0891x_{2}+0.0733x_{3}+0.0094x_{1}x_{2}+0.0086x_{1}x_{3}+0.0109x_{2}x_{3}$$

(9) $$y_{Me}=1.2024+0.1209x_{1}+0.1656x_{2}+0.1326x_{3}+0.0161x_{1}x_{2}+0.0141x_{1}x_{3}+0.0184x_{2}x_{3}$$

Among the model terms, extraction time (x_2_) had the most significant effect on CA yield, with extremely high F-values ranging from 623.00 to 931.69 (*p* < 0.00001), indicating it was the dominant factor influencing extraction efficiency. This was followed by extraction temperature (x_3_), which also showed a strong and significant impact (F = 422.26–807.50, *p* < 0.00001). Ethanol concentration (x_1_) was also statistically significant, with F-values between 269.55 and 512.03 (*p* < 0.00001).

Notably, the interaction between extraction time and temperature (x_2_·x_3_) had a meaningful influence on CA content, with F-values between 9.27 and 12.94 (*p* < 0.01593), suggesting a synergistic effect. In contrast, the three-way interaction (x_1_·x_2_·x_3_) was not statistically significant (F = 0.1052–0.6470, *p* > 0.05), indicating it had little to no effect on the extraction process.

**Figure 7:**
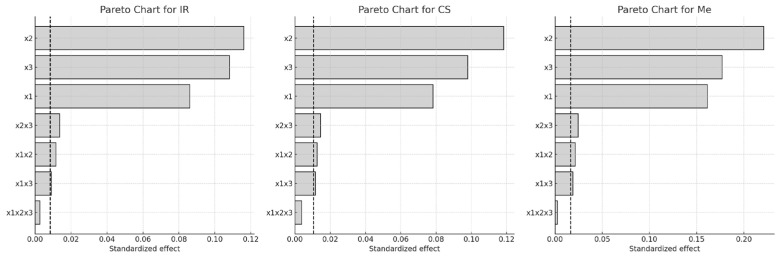
Pareto charts of factors influence on CA content in extracts of different grape stems: x_1_, ethanol concentration; x_2_, extraction time; x_3_, extraction temperature (Dashed line indicates the significance limit for standardized effects).

**Figure 8:**
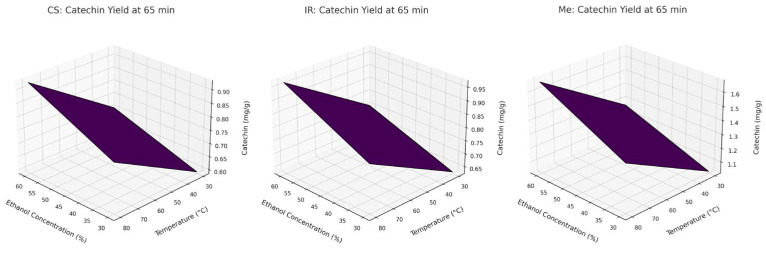
Response-surface plots for CA extraction.

These effects of factors on the CA extraction process are shown in Figure 7 using a Pareto diagram and Figure 8 using a 3D graph.

**Figure 9:**
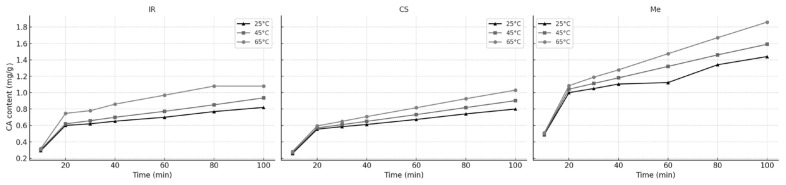
Variation in CA contents in extracts during the extraction process at (▲) 25 °C; (▪) 45 °C; and (•) 65 °C; for Ir, CS, and Me grape stems.

Figure 9 illustrates the increase in CA content over time during extraction with 60% ethanol from different grape stem varieties at varying temperatures. The resulting curves display a typical extraction profile for plant-derived compounds, consistent with trends observed in our previous experiments.

Across all grape stem varieties and extraction temperatures (25 °C, 45 °C, and 65 °C), the unsteady-state diffusion model provided an excellent fit to the experimental data. The coefficients of determination (R^2^) ranged from 0.9852 to 0.9992, while the root mean square (RMS) errors remained low, between 0.62 and 2.87 (Table 10). These results confirm that the derived empirical equations are suitable for predicting system responses within the studied parameter ranges.

The calculated activation energies for catechin extraction were all positive, indicating that the process is endothermic. The Ea values were 8.54 kJ/mol for Italian Riesling (IR), 8.39 kJ/mol for Cabernet Sauvignon (CS), and 7.85 kJ/mol for Merlot (Me). Regardless of grape stem variety, the extraction rate constant increased with temperature, confirming the temperature dependence of the process.

The thermodynamic parameters for CA extraction from grape stems are summarized in Table 10. The enthalpy change (ΔH°) values ranged from 26.29 to 35.25 kJ/mol across the different grape varieties, confirming the endothermic nature of the process. Similarly, the entropy change (ΔS°) was positive for all samples, with values ranging from 95.15 to 131.57 J/mol·K. Merlot (Me) stems exhibited the highest ΔS°, indicating a greater degree of disorder and further supporting the irreversibility of the process.

The Gibbs free energy change (ΔG°) values were negative in all cases, ranging from –2.04 to –9.22 kJ/mol, demonstrating that the extraction of CA is thermodynamically feasible and spontaneous. The most negative ΔG° values were observed in extracts from Merlot stems, indicating the highest spontaneity.

Overall, the most favorable extraction conditions for CA were observed at 65 °C from Merlot stems, which exhibited the lowest activation energy, highest kinetic and equilibrium constants (k and K), and the most negative ΔG°, confirming the efficiency and thermodynamic favorability of the process.

### 2.5. Characterization of Extracts Obtained Under Optimal Conditions

The composition and antioxidant activity of grape stem extracts obtained under optimal conditions (60% ethanol, 65 °C, and 80 min) are presented in Table 11. Among the varieties tested, Merlot (Me) showed the highest total phenolic (72.22 mg GAE/g) and flavonoid content (62.83 mg CAE/g), along with the highest levels of Q-gluc (1.098 mg/g), Q-glc (0.409 mg/g), and catechin (1.860 mg/g).

Merlot also exhibited the strongest antioxidant activity across all assays (DPPH, ABTS, FRAP, CUPRAC, TRP), confirming a strong correlation between phenolic content and antioxidant potential. These results are consistent with literature data, where Merlot stems have been reported as rich in bioactive flavonoids [30,31].

Cabernet Sauvignon (CS) showed intermediate values, while Italian Riesling (IR) had the lowest, supporting the conclusion that Merlot stems are the most promising source of natural antioxidants for potential food, pharmaceutical, or cosmetic applications [32,33].

From an applied perspective, these optimized extractions could be scaled for industrial use, where grape stems represent a low-cost raw material generated in large volumes by wineries. Developing stable antioxidant extracts from this by-product may offer the food, nutraceutical, and cosmetic industries a natural alternative to synthetic antioxidants, while simultaneously reducing waste management challenges for the wine sector. When compared to other well-known plant sources such as tea leaves [34], grape stems generally show lower absolute levels of catechins and quercetin derivatives. However, their abundance as a winery by-product makes them a sustainable and attractive alternative source for these bioactive compounds.

## 3. Materials and Methods

### 3.1. Samples

Seven well-known international and regional grape varieties were included in this study (Table 12): four red varieties—‘Cabernet Sauvignon’ (CS), ‘Merlot’ (Me), ‘Prokupac’ (Pr), and ‘Vranac’ (Vr); and three white varieties— ‘Italian Riesling’ (IR), ‘Smederevka’ (Sm), and ‘Župljanka’ (Žu).

The samples of grape were collected from vineyards in the south-eastern region of Serbia. The stems were manually separated from the grape clusters, thoroughly washed with distilled water and air-dried for 20 days at room temperature in the dark. The dried plant material was then stored in paper bags under cool, dry and dark conditions until analysed. Prior to extraction, the stems were ground with a hammer mill and sieved through a 6 mm sieve to ensure a uniform particle size.

### 3.2. Initial Content of Individual Phenolic Compounds (q_0_)

A total of 2.0 g of grape stem material was weighed into a 250 mL Erlenmeyer flask with a ground-glass stopper and extracted with 100 mL of 60% (*v*/*v*) aqueous ethanol. To minimize possible catechin degradation, the solvent was freshly prepared, and the extraction performed under reduced exposure to air and light. Maceration was performed for 120 min. The extract was then separated from the plant residues by filtration through Whatman No. 1 filter paper (Whatman, Maidstone, UK). The solid residues were re-extracted twice using fresh solvent, with each subsequent maceration lasting 30 min. All three extracts were combined.

The combined extracts were concentrated under reduced pressure at 45 °C using a rotary evaporator (BUCHI Rotavapor R-200, BÜCHI Labortechnik AG, Flawil, Switzerland). The dried crude extracts were then re-dissolved in 60% ethanol prior to analysis. All experiments were conducted in triplicate.

Quantification of phenolic compounds was carried out using an HPLC-DAD method. The initial concentrations of Q-gluc, Q-glc, and CA in the stems of Italian Riesling (IR), Cabernet Sauvignon (CS), and Merlot (Me), respectively, were as follows (in mg/g dry weight): Q-gluc: 0.585 ± 0.012 (IR), 0.650 ± 0.010 (CS), 1.320 ± 0.024 (Me); Q-glc: 0.123 ± 0.004 (IR), 0.172 ± 0.005 (CS), 0.478 ± 0.008 (Me); CA: 1.182 ± 0.020 (IR), 1.215 ± 0.018 (CS), 1.988 ± 0.024 (Me).

### 3.3. HPLC-DAD Analysis of Extracts

Phenolic compounds were identified and quantified using an Agilent 1200 Series HPLC-DAD system (Agilent Technologies, Santa Clara, CA, USA) with gradient elution on a C18 column, as previously described by Mitić et al. (2024) [13]. Compounds were identified by comparing retention times and UV spectra with standards, and quantification was based on calibration curves.

The calibration curve, correlation coefficient (R^2^), limit of detection (LOD), and limit of quantification (LOQ) are presented in Table 13.

The concentrations of the constituents in the samples were calculated using the equations obtained from calibration curves prepared for each standard. For compounds without corresponding standards, quantification was performed using calibration curves of structurally related compounds. Specifically, caffeic acid was used as the standard for trans-caftaric acid (caffeoyltartaric acid), luteolin for luteolin-glucoside, and quercetin-3-O-glucoside for quercetin glucuronide. The results were expressed as mg/g of dry weight.

### 3.4. Spectrophotometric Analysis

Total phenolic content (TP) was determined using the Folin–Ciocalteu method and expressed as mg gallic acid equivalents per g dry weight, while total flavonoid content (TF) was measured using the aluminum chloride colorimetric method and reported as mg catechin equivalents per g dry weight. Antioxidant activity of the extracts was evaluated using four complementary assays: DPPH, ABTS, FRAP, and CUPRAC, which collectively assess radical scavenging capacity and reducing power. All spectrophotometric methods were performed as described in previous studies and adapted for plant-based extracts [13,31].

### 3.5. Optimization of the Extraction Procedure Using Experimental Design and Statistical Analysis

In the initial phase, extraction experiments were conducted under varying conditions of ethanol concentration, extraction time, and temperature, using a fixed solvent-to-solid ratio of 30:1 (mL/g). The experimental conditions were defined based on a 2^3^ full factorial design, as shown in Table 1, Table 4 and Table 7. Maceration was applied as the extraction method for isolating Q-gluc, Q-glc, and CA from grape stems.

According to the design, 2.0 g of stem material was subjected to extraction using different combinations of the selected variables. Extractions were performed in a temperature-controlled water bath. Following extraction, the mixtures were filtered using Whatman filter paper 6 μm pore size (Whatman, Cytiva, Marlborough, MA, USA), and the resulting filtrates were collected and stored in sealed flasks for analysis.

The experimental data were modeled using a first-order polynomial equation (Equation (10)), obtained via linear regression. This model described the relationship between the independent variables—x_1_ (ethanol concentration), x_2_ (extraction time), and x_3_ (temperature)—and the response variables: y_1_ (Q-gluc content), y_2_ (Q-glc content), and y_3_ (CA content).

(10) $$y=b_{o}+b_{1}x_{1}+b_{2}x_{2}+b_{3}x_{3}+b_{12}x_{1}x_{2}+b_{13}x_{1}x_{3}+b_{23}x_{2}x_{3}+b_{123}x_{1}x_{2}x_{3}$$

where $x_{1}$, $x_{2}$ and $x_{3}$, were identified to be ethanol concentration, time and temperature, respectively. The equation terms, such as $x_{1}x_{2}$, $x_{1}x_{3}$, $x_{2}x_{3}$ describe the interaction of two independent variables, and $x_{1}x_{2}x_{3}$ establish the interaction of three independent variables. $b_{o}$ is the constant regression coefficient, $b_{i}$ are the linear regression coefficient, $b_{ij}$ and $b_{ijk}$ are the regression coefficients of 2- and 3-factor interactions, respectively.

### 3.6. Kinetics of Extraction

Grape stem samples and extraction solvent were combined at a solvent-to-solid ratio of 30:1 (mL/g) in a series of 250 mL Erlenmeyer flasks (all extractions were performed with minimal exposure to light to avoid degradation of phenolics). Maceration was conducted at controlled temperatures of 25 ± 0.1 °C, 45 ± 0.1 °C, and 65 ± 0.1 °C, with extraction times of 10, 20, 30, 40, 60, 80, and 100 min. At each time point, one flask was removed from the water bath, and the extract was separated from the plant material by vacuum filtration The concentrations of Q-gluc, Q-glc, and CA in the liquid phase were quantified using the HPLC-DAD method.

### 3.7. Kinetic Model-Unsteady-State Diffusion Model

The basic equation of unsteady-state diffusion model is as follows [35,36]:

(11) $$q_{i}/q_{o}=\left(1-b\right)e^{-kt}$$

(12) $$ln\left(q_{i}/q_{o}\right)=ln\left(1-b\right)-kt$$

where $q_{i}$ is the Q-gluc, Q-glc or CA contents in the grape stems during extractione (mg/g), $q_{o}$ is the Q-gluc, Q-glc or CA contents initially present in the grape stems (mg/g), k is slow extraction coefficient of the unsteady-state diffusion model (1/min), and b is the washing coefficient of the unsteady-state diffusion model.

### 3.8. Validity of the Kinetic Model

The suitability of the applied kinetic model was evaluated using the coefficient of determination (R^2^) and the root mean square error (RMS), as proposed by Kitanović et al. (2008) [37], and calculated using Equations (13) and (14):

(13) $$R^{2}=\frac{\sum_{i=1}^{N}{\left(q_{ex}-q_{pre}\right)}^{2}}{\sum_{i=1}^{N}{\left(q_{ex}-q_{m}\right)}^{2}}$$

(14) $$RMS=\sqrt{\frac{1}{N}\sum_{i=1}^{N}\left(\frac{q_{ex}-q_{pre}}{q_{ex}}\right)}$$

where $q_{ex}$ and $q_{pre}$ are the experimental and predicted values of the phenolic compounds, $q_{m}$ the mean value of phenolic compounds, and N is the number of the experimental runs.

A higher R^2^ value and a lower RMS value indicate a better fit of the model to the experimental data.

### 3.9. Thermodynamic Study

The Arrhenius equation was used for the calculation of the activation energy, which was used to describe the relationship between the k slow extraction coefficient of the unsteady-state diffusion model and the temperature. It is expressed in Equation (15), and can be rearranged as a linear Equation (16) [38]:

(15) $$k=Ae^{{-E}_{a}/RT}$$

(16) $$lnk=lnA-\frac{E_{a}}{R}\frac{1}{T}$$

where E_a_ is the activation energy (kJ/mol), R is the gas constant, and T is the absolute temperature (K) [39].

Thermodynamic parameters ($∆H^{o},$ $∆S^{o}$ and $∆G^{o}$) were estimated using Equations (17) and (18) [40,41]:

(17) $$∆G^{o}=-RTlnK_{e}$$

(18) $$lnK_{e}=-\frac{∆H^{o}}{R}\frac{1}{T}+\frac{∆S^{o}}{R}$$

(19) $$K_{e}=\frac{m_{L}}{m_{S}}$$

where K_e_ is equilibrium constant, m_L_ is the amount of chlorogenic acid, ferulic acid or sinapic acid in liquid at equilibrium temperature (T), m_S_ is the amount of these acids in solid at T, $∆H^{o}$ (kJ/mol) is enthalpy change, $∆S^{o}$ (J/mol K) is entropy change and $∆G^{o}$ (kJ/mol) is the standard Gibbs free energy change (kJ/mol). $∆H^{o}$ and $∆S^{o}$ can be determined using the slope and y-intercept of the plot of lnK_e_ versus 1/T based on Equation (18).

## 4. Conclusions

This study applied a full factorial 2^3^ design to optimize extraction of Q-gluc, Q-glc, and CA from grape stems. Temperature, time, and ethanol concentration were identified as the main factors influencing efficiency, with kinetic modeling confirming a diffusion-driven process. Among the tested varieties, Merlot stems consistently provided the highest yields, making them the most suitable source of these phenolic compounds. These findings not only clarify the extraction dynamics of grape stem phenolics but also highlight the potential of grape stems as an underutilized by-product that can be transformed into high-value raw material. This approach yields an extract with the highest combined content of all three target phenolic compounds, making it the most promising raw material for further application in functional food, nutraceutical, or pharmaceutical formulations. Nonetheless, some limitations should be acknowledged. The variability of raw materials between vintages and regions, as well as the scalability of the optimized process to industrial level, remain open challenges. A detailed economic assessment and a more complete green chemistry evaluation would also be valuable to confirm the feasibility of this approach in real-world applications. Thus, this study provides both methodological insights and practical guidance for valorizing grape stems within sustainable and circular economy strategies.

## Figures and Tables

**Table 1:** Individual phenolic composition in seven different varieties of grape stems.

Compound/ Sample	IR	Sm	Žu	Cs	Me	Pr	Vr
CA	1.182 ± 0.020 ^1^c	0.930 ± 0.012 de	0.608 ± 0.009 f	1.215 ± 0.030 b	1.988 ± 0.032 a	0.895 ± 0.040 e	0.958 ± 0.038 d
ECA	0.022 ± 0.002 c	0.035 ± 0.002 a	0.012 ± 0.002 e	0.026 ± 0.003 b	0.020 ± 0.002 c	0.016 ± 0.006 d	0.020 ± 0.002 c
Q-ru	0.046 ± 0.010 c	0.110 ± 0.008 a	0.036 ± 0.010 cd	0.020 ± 0.001 d	0.024 ± 0.003 d	0.108 ± 0.012 a	0.092 ± 0.008 b
Q-gluc	0.585 ± 0.042 c	0.552 ± 0.020 c	0.550 ± 0.018 c	0.650 ± 0.032 b	1.320 ± 0.028 a	0.480 ± 0.016 d	0.520 ± 0.026 d
Q-glc	0.123 ± 0.005 e	0.072 ± 0.010 g	0.110 ± 0.009 f	0.172 ± 0.020 d	0.478 ± 0.022 a	0.250 ± 0.012 b	0.230 ± 0.010 c
Km	0.018 ± 0.002 b	0.011 ± 0.002 d	0.010 ± 0.001 d	0.015 ± 0.002 cb	0.018 ± 0.002 b	0.020 ± 0.003 ab	0.022 ± 0.004 a
L-glc	0.398 ± 0.015 b	0.270 ± 0.012 c	0.060 ± 0.002 f	0.105 ± 0.008 e	0.452 ± 0.015 a	0.170 ± 0.010 d	0.182 ± 0.016 d
Caf	0.094 ± 0.012 c	0.028 ± 0.001 d	0.024 ± 0.006 d	0.138 ± 0.010 a	0.110 ± 0.010 b	0.090 ± 0.012 c	0.090 ± 0.012 c
Caff	0.273 ± 0.028 b	0.392 ± 0.022 a	0.025 ± 0.006 e	0.105 ± 0.012 d	0.108 ± 0.018 d	0.032 ± 0.008 e	0.210 ± 0.028 c
Sy	0.118 ± 0.013 a	0.063 ± 0.010 c	0.010 ± 0.002 f	0.072 ± 0.008 b	0.040 ± 0.006 d	0.022 ± 0.006 e	0.020 ± 0.004 ef
Cm	0.242 ± 0.026 a	0.180 ± 0.012 d	0.020 ± 0.002 e	0.202 ± 0.010 c	0.225 ± 0.013 b	0.180 ± 0.020 d	0.175 ± 0.020 d
∑	3.101	2.643	1.465	2.720	4.783	2.263	2.519

Italian Riesling (IR); Smederevka (Sm); Župljanka (Žu); Cabernet Sauvignon (CS); Merlot (Me); Prokupac (Pr); and Vranac (Vr); CA—(+)-catechin; ECA—epicatechin; Q-ru—Quercetin-3-rutinoside; Q-gluc—Quercetin-3-glucuronide; Q-glc—Quercetin-glucoside; Km- Kaemferol; L-glc—Luteolin glucoside; Caf—t-Caftaric acid; Caff—Caffeic acid; Sy—Syringic acid; Cm—p-Coumaric acid. ^1^ The results are present as mean ± SD (*n* = 3), and values in each row with different letters are significantly different (p < 0.05).

**Table 2:** Experimental values and coded levels of the independent variables used for the Experimental design with the observed responses values for Q-3-gluc.

No	Design Matrix			IR		CS		Me	
	x_1_ (%, *v*/*v*)	x_2_ (min)	x_3_ (°C)	q_ex_ ^2^ (mg/g)	q_pre_ ^3^ (mg/g)	q_ex_ (mg/g)	q_pre_ (mg/g)	q_ex_ (mg/g)	q_pre_ (mg/g)
1	30(−1)	30(−1)	25(−1)	0.217 ± 0.008 ^1^	0.215	0.221 ± 0.006	0.222	0.431 ± 0.010	0.433
2	60(+1)	30(−1)	25(−1)	0.286 ± 0.011	0.288	0.294 ± 0.008	0.293	0.581 ± 0.010	0.579
3	30(−1)	80(+1)	25(−1)	0.265 ± 0.006	0.263	0.278 ± 0.006	0.276	0.556 ± 0.012	0.553
4	60(+1)	80(+1)	25(−1)	0.344 ± 0.008	0.341	0.370 ± 0.011	0.372	0.751 ± 0.012	0.753
5	30(−1)	30(−1)	65(+1)	0.305 ± 0.006	0.307	0.287 ± 0.005	0.285	0.565 ± 0.009	0.662
6	60(+1)	30(−1)	65(+1)	0.366 ± 0.012	0.364	0.382 ± 0.011	0.384	0.759 ± 0.012	0.761
7	30(−1)	80(+1)	65(+1)	0.338 ± 0.005	0.342	0.364 ± 0.006	0.365	0.730 ± 0.010	0.733
8	60(+1)	80(+1)	65(+1)	0.440 ± 0.007	0.438	0.482 ± 0.012	0.483	0.984 ± 0.008	0.982

x_1_—Ethanol concentration; x_2_—Extraction time; x_3_—Extraction temperature; IR—Italian Riesling; CS—Cabernet Sauvignon; Me—Merlot; ^1^ Mean values (*n* = 3) *±* standard deviation; ^2^ Experimental results; ^3^ Predicted results.

**Table 3:** Results of the analysis variance for Q-gluc.

Grape Stems	SOV	x_1_	x_2_	x_3_	x_1_x_2_	x_1_x_3_	x_2_x_3_	x_1_x_2_x_3_	Error	Total
IR	SS ^1^	0.012090	0.005671	0.014196	0.000325	0.000028	0.000001	0.000120	0.000176	0.032607
	Df ^2^	1	1	1	1	1	1	1	8	15
	MS ^3^	0.012090	0.005671	0.014196	0.000325	0.000028	0.000001	0.000120	0.000022	0.0021738
	F-Value	549.54	257.77	645.27	14.772	1.2727	0.04545	5.4545		
	*p*-Value	<0.00001	<0.00001	<0.00001	0.004923	0.291955	0.8366	0.047751		
	R^2^ = 0.9946; R_adj_^2^ = 0.9898; CV(%) = 1.46.									
CS	SS	0.017861	0.012012	0.015488	0.000221	0.000288	0.000242	0.000006	0.000320	0.046438
	Df	1	1	1	1	1	1	1	8	15
	MS	0.017861	0.012012	0.015488	0.000221	0.000288	0.000242	0.000006	0.000040	0.003096
	F-Value	446.52	300.30	387.20	5.5250	7.2000	6.0500	0.1500		
	*p*-Value	<0.00001	<0.00001	<0.00001	0.046643	0.027784	0.039339	0.708635		
	R^2^ = 0.9931; R_adj_^2^ = 0.9871; CV(%) = 1809.									
Me	SS	0.078606	0.058653	0.064620	0.001378	0.001326	0.001128	0.000028	0.001304	0.21944
	Df	1	1	1	1	1	1	1	8	15
	MS	0.078606	0.058653	0.064620	0.001378	0.001326	0.001128	0.000028	0.000163	0.014629
	F-Value	482.24	359.83	396.44	8.4539	8.1349	6.9200	0.1717		
	*p*-Value	<0.00001	0.001146	<0.00001	0.019663	0.021407	0.030151	0.689493		
	R^2^ = 0.9940; R_adj_^2^ = 0.9888; CV(%) = 1.91.									

x_1_—Ethanol concentration; x_2_—Extraction time; x_3_—Extraction temperature; SOV: source of variation; ^1^: sum of squares; ^2^: degree of freedom; ^3^: mean of square.

**Table 4:** Values of kinetics and thermodynamic parameters for Q-gluc extraction.

Stems	IR			CS			Me		
	25 °C	45 °C	65 °C	25 °C	45 °C	65 °C	25 °C	45 °C	65 °C
b	0.772	0.777	0.779	0.758	0.764	0.772	0.741	0.751	0.763
k (1/min)	1.52·10^−3^	2.00·10^−3^	2.55·10^−3^	1.67·10^−3^	2.18·10^−3^	2.82·10^−3^	2.02·10^−3^	2.42·10^−3^	3.03·10^−3^
RMS	1.65	1.83	1.86	1.82	0.69	1.15	1.18	1.76	1.45
R^2^	0.9940	0.9928	0.9930	0.9930	0.9993	0.9952	0.9948	0.9932	0.9938
E_a_ (kJ/mol)	10.83			10.96			8.47		
K	1.58	2.54	4.00	2.09	2.56	5.22	1.64	2.69	6.06
ΔH° (kJ/mol)	19.47			22.08			24.17		
ΔS° (J/molK)	69.11			79.76			84.96		
ΔG° (kJ/mol)	−1.12	−2.50	−3.88	−1.68	−3.28	−4.55	−1.72	−3.45	−4.88

b—washing coefficient; RMS—root mean square; R^2^—coefficient of determination.

**Table 5:** Experimental values and coded levels of the independent variables used for the Experimental design with the observed responses values for Q-glc.

No	Design Matrix			IR		CS		Me	
	x_1_ (%, *v*/*v*)	x_2_ (min)	x_3_ (°C)	q_ex_ ^2^ (mg/g)	q_pre_ ^3^ (mg/g)	q_ex_ (mg/g)	q_pre_ (mg/g)	q_ex_ (mg/g)	q_pre_ (mg/g)
1	30(−1)	30(−1)	25(−1)	0.045 ± 0.003 ^1^	0.042	0.066 ± 0.003	0.062	0.177 ± 0.014	0.176
2	60(+1)	30(−1)	25(−1)	0.058 ± 0.001	0.059	0.084 ± 0.001	0.085	0.222 ± 0.016	0.223
3	30(−1)	80(+1)	25(−1)	0.055 ± 0.002	0.056	0.082 ± 0.001	0.083	0.227 ± 0.012	0.228
4	60(+1)	80(+1)	25(−1)	0.071 ± 0.004	0.072	0.105 ± 0.003	0.107	0.277 ± 0.011	0.275
5	30(−1)	30(−1)	65(+1)	0.058 ± 0.002	0.060	0.087 ± 0.003	0.088	0.241 ± 0.009	0.237
6	60(+1)	30(−1)	65(+1)	0.076 ± 0.002	0.076	0.111 ± 0.001	0.113	0.303 ± 0.014	0.296
7	30(−1)	80(+1)	65(+1)	0.072 ± 0.003	0.074	0.108 ± 0.002	0.110	0.309 ± 0.012	0.313
8	60(+1)	80(+1)	65(+1)	0.092 ± 0.004	0.090	0.139 ± 0.002	0.134	0.386 ± 0.008	0.382

x_1_—ethanol concentration; x_2_—extraction time; x_3_—extraction temperature; IR—Italian Riesling; CS—Cabernet Sauvignon; Me—Merlot; ^1^ Mean values (*n* = 3) *±* standard deviation; ^2^ Experimental results; ^3^ Predicted results.

**Table 6:** Results of the analysis variance for Q-glc.

Grape Stems	SOV	x_1_	x_2_	x_3_	x_1_x_2_	x_1_x_3_	x_2_x_3_	x_1_x_2_x_3_	Error	Total
IR	SS ^1^	0.000561	0.000351	0.000595	0.000003	0.000010	0.000006	0.000001	0.000048	0.001575
	Df ^2^	1	1	1	1	1	1	1	8	15
	MS ^3^	0.000561	0.000351	0.000595	0.000003	0.000010	0.000006	0.000001	0.000006	0.000105
	F-Value	93.500	58.500	99.166	0.5000	1.6666	1.0000	0.1666		
	*p*-Value	0.000011	0.00006	<0.00001	0.499576	0.232768	0.34594	0.693857		
	R^2^ = 0.9635; R_adj_^2^ = 0.9428; CV(%) = 3.71.									
CS	SS	0.001152	0.000924	0.001458	0.000018	0.000024	0.000018	0.000001	0.000104	0.003699
	Df	1	1	1	1	1	1	1	8	15
	MS	0.001152	0.000924	0.001458	0.000018	0.000024	0.000018	0.000001	0.000013	0.0002466
	F-Value	88.615	71.077	11.215	1.3846	1.8461	1.3846	0.07692		
	*p*-Value	0.000013	0.000030	0.010097	0.273142	0.211305	0.73142	0.788574		
	R^2^ = 0.9719; R_adj_^2^ = 0.9473; CV(%) = 3.69.									
Me	SS	0.006844	0.008192	0.014112	0.000050	0.000242	0.000264	0.000012	0.000352	0.030068
	Df	1	1	1	1	1	1	1	8	15
	MS	0.006844	0.008192	0.014112	0.000050	0.000242	0.000264	0.000012	0.000044	0.002004
	F-Value	155.54	186.18	320.72	1.1363	5.5000	6.0000	0.2727		
	*p*-Value	<0.00001	<0.00001	<0.00001	0.317546	0.047032	0.039969	0.615674		
	R^2^ = 0.9883; R_adj_^2^ = 0.9780; CV(%) = 2.47.									

x_1_—Ethanol concentration; x_2_—Extraction time; x_3_—Extraction temperature; SOV—source of variation; ^1^ sum of squares; ^2^ degree of freedom; ^3^ mean of square.

**Table 7:** Values of kinetics and thermodynamic parameters for Q-glc extraction.

Stems	IR			CS			Me		
	25 °C	45 °C	65 °C	25 °C	45 °C	65 °C	25 °C	45 °C	65 °C
b	0.770	0.773	0.776	0.780	0.784	0.791	0.767	0.769	0.782
k (1/min)	1.53·10^−3^	2.07·10^−3^	2.78·10^−3^	1.48·10^−3^	2.25·10^−3^	2.98·10^−3^	1.57·10^−3^	2.39·10^−3^	2.87·10^−3^
RMS	1.16	1.34	1.89	0.86	1.08	1.46	0.63	1.14	3.02
R^2^	0.9988	0.9980	0.9934	0.9989	0.9982	0.9979	0.9992	0.9984	0.9862
E_a_ (kJ/mol)	12.49			11.39			12.68		
K	1.86	2.84	7.20	2.31	4.37	13.33	2.02	3.05	7.24
ΔH° (kJ/mol)	28.17			36.58			26.53		
ΔS° (J/molK)	99.23			129.18			84.47		
ΔG° (kJ/mol)	−1.40	−3.38	−5.37	−1.92	−4.50	−7.08	−1.62	−3.51	−5.4055

b—washing coefficient; RMS—root mean square; R^2^—coefficient of determination.

**Table 8:** Experimental values and coded levels of the independent variables used for the Experimental design with the observed responses values for CA.

No	Design Matrix			IR		CS		Me	
	x_1_ (%, *v*/*v*)	x_2_ (min)	x_3_ (°C)	q_ex_ ^2^ (mg/g)	q_pre_ ^3^ (mg/g)	q_ex_ (mg/g)	q_pre_ (mg/g)	q_ex_ (mg/g)	q_pre_ (mg/g)
1	30(−1)	30(−1)	25(−1)	0.500 ± 0.016 ^1^	0.502	0.481 ± 0.010	0.484	0.830 ± 0.020	0.832
2	60(+1)	30(−1)	25(−1)	0.602 ± 0.015	0.600	0.568 ± 0.008	0.565	1.015 ± 0.018	1.013
3	30(−1)	80(+1)	25(−1)	0.640 ± 0.016	0.638	0.625 ± 0.012	0.622	1.096 ± 0.022	1.094
4	60(+1)	80(+1)	25(−1)	0.769 ± 0.018	0.771	0.738 ± 0.014	0.741	1.338 ± 0.020	1.340
5	30(−1)	30(−1)	65(+1)	0.632 ± 0.010	0.630	0.595 ± 0.009	0.592	1.034 ± 0.009	1.032
6	60(+1)	30(−1)	65(+1)	0.753 ± 0.012	0.755	0.705 ± 0.011	0.707	1.268 ± 0.022	1.267
7	30(−1)	80(+1)	65(+1)	0.805 ± 0.015	0.807	0.770 ± 0.016	0.773	1.366 ± 0.018	1.368
8	60(+1)	80(+1)	65(+1)	0.969 ± 0.017	0.967	0.929 ± 0.018	0.926	1.672 ± 0.021	1.670

x_1_—ethanol concentration; x_2_—extraction time; x_3_—extraction temperature; IR—Italian Riesling; CS—Cabernet Sauvignon; Me—Merlot; ^1^ Mean values (*n* = 3) *±* standard deviation; ^2^ Experimental results; ^3^ Predicted results.

**Table 9:** Results of the analysis variance for catechin.

Grape Stems	SOV	x_1_	x_2_	x_3_	x_1_x_2_	x_1_x_3_	x_2_x_3_	x_1_x_2_x_3_	Error	Total
IR	SS ^1^	0.033282	0.060552	0.052488	0.000612	0.000364	0.000841	0.000032	0.000520	0.148691
	Df ^2^	1	1	1	1	1	1	1	8	15
	MS ^3^	0.033282	0.060552	0.052488	0.000612	0.000364	0.000841	0.000032	0.000065	0.009913
	F-Value	512.03	931.69	807.50	9.4153	5.6000	12.938	0.4923		
	*p*-Value	<0.00001	<0.00001	<0.00001	0.015384	0.045499	0.007013	0.5028		
	R^2^ = 0.9965; R_adj_^2^ = 0.9934; CV(%) = 1.14.									
CS	SS	0.027495	0.063546	0.043071	0.000703	0.000595	0.000946	0.000066	0.000816	0.137238
	Df	1	1	1	1	1	1	1	8	15
	MS	0.07495	0.063546	0.043071	0.000703	0.000595	0.000946	0.000066	0.000102	0.009149
	F-Value	269.55	623.00	422.26	6.8921	5.8333	9.2745	0.6470		
	*p*-Value	<0.00001	<0.00001	<0.00001	0.030400	0.042163	0.015932	0.444431		
	R^2^ = 0.9940; R_adj_^2^ = 0.9888; CV(%) = 1.49.									
Me	SS	0.116886	0.219453	0.140715	0.002080	0.001596	0.002701	0.000028	0.002126	0.485585
	Df	1	1	1	1	1	1	1	8	15
	MS	0.116886	0.219453	0.140715	0.002080	0.001596	0.002701	0.000028	0.000263	0.032372
	F-Value	439.42	825.01	529.00	7.8195	6.0000	10.154	0.1052		
	*p*-Value	<0.00001	<0.00001	<0.00001	0.023329	0.039969	0.01287	0.753995		
	R^2^ = 0.9956; R_adj_^2^ = 0.9918; CV(%) = 1.35.									

x_1_—Ethanol concentration; x_2_—Extraction time; x_3_—Extraction temperature; SOV: source of variation; ^1^ sum of squares; ^2^ degree of freedom; ^3^ mean of square.

**Table 10:** Values of kinetics and thermodynamic parameters for CA extraction.

Stems	IR			CS			Me		
	25 °C	45 °C	65 °C	25 °C	45 °C	65 °C	25 °C	45 °C	65 °C
b	0.786	0.791	0.803	0.761	0.763	0.771	0.781	0.791	0.792
k (1/min)	1.73·10^−3^	2.22·10^−3^	2.60·10^−3^	1.99·10^−3^	2.49·10^−3^	2.79·10^−3^	1.97·10^−3^	2.28·10^−3^	2.87·10^−3^
RMS	0.62	0.67	2.87	0.57	1.63	1.62	2.86	1.01	2.18
R^2^	0.9992	0.9990	9890	0.9992	0.9948	0.9938	0.9852	0.9983	0.9888
E_a_ (kJ/mol)	8.54			8.39			7.85		
K	2.44	4.01	12.13	2.42	3.58	8.57	2.91	4.88	15.84
ΔH° (kJ/mol)	33.42			26.29			35.25		
ΔS° (J/molK)	118.99			95.15			131.57		
ΔG° (kJ/mol)	−2.04	−4.42	−6.80	−2.06	−3.01	−5.87	−3.96	−6.59	−9.22

b—washing coefficient; RMS—root mean square; R^2^—coefficient of determination.

**Table 11:** Phenolic composition and antioxidant activity of grape stem extracts under optimal extraction conditions.

	IR	Cs	Me
TP	58.20 ± 0.18	69.83 ± 0.30	72.22 ± 0.28
TF	46.38 ± 0.12	55.30 ± 0.22	62.83 ± 0.20
Q-gluc	0.465 ± 0.012	0.538 ± 0.012	1.098 ± 0.020
Q-glc	0.102 ± 0.080	0.154 ± 0.008	0.409 ± 0.010
CA	1.080 ± 0.032	1.030 ± 0.018	1.860 ± 0.032
DPPH	0.678 ± 0.025	0.692 ± 0.052	0.715 ± 0.048
ABTS	0.702 ± 0.030	0.738 ± 0.038	0.772 ± 0.062
FRAP	0.632 ± 0.018	0.685 ± 0.042	0.702 ± 0.048
CUPRAC	0.832 ± 0.020	0.870 ± 0.050	0.953 ± 0.082
TRP	0.485 ± 0.022	0.492 ± 0.028	0.536 ± 0.038

TP: total phenolics (mg gallic acid equivalents per g dry weight); TF: total flavonoids (mg catechin equivalents per g dry weight); FRAP: expressed as mmol Fe^2+^ equivalents per g dry weight; TRP: expressed as mg ascorbic acid equivalents (AAE) per g dry weight.

**Table 12:** Tested grape varieties.

No	Grape Varieties	Abreviation	Varieties Characteristics	Origin Country
1	‘Cabernet Sauvignon’	CS	*Vitis vinifera* L., international, red wine variety	France
2	‘Merlot’	Me	*Vitis vinifera* L., international, red wine variety	France
3	‘Prokupac’	Pr	*Vitis vinifera* L., autochthonous, red wine variety	Serbia
4	‘Vranac’	Vr	*Vitis vinifera* L., autochthonous, red wine variety	Montenegro
5	‘Italian Riesling’	IR	*Vitis vinifera* L., international, white wine variety	Italy
6	‘Smederevka’	Sm	*Vitis vinifera* L., autochthonous, white wine variety	Serbia
7	‘Župljanka’	Žu	*Vitis vinifera* L., white wine variety new created: Prokupac x Pinot Noir	Serbia

**Table 13:** Validation parameters for 10 phenolic compounds used for HPLC-DAD analysis.

Compound	Calibration Curve	(R^2^)	LOD ^1^ (µg/mL)	LOQ ^2^ (µg/mL)
CA	y=4628.36x+0.97	0.9996	0.33	1.10
ECA	y=4785.17x−0.18	0.9998	0.30	1.00
Q-ru	*y* =4879.79*x* − 5.55	0.9996	0.39	1.18
Q-glc	y=5209.08x−1.05	0.9996	0.48	1.45
Q	*y* =8143.54*x* + 5.62	0.9999	0.52	1.57
Km	*y* =18,921.26*x* + 1.82	0.9999	0.55	1.67
L	*y* =3542.67*x* + 1.81	0.9997	0.63	1.93
Caff	*y* =33,621.18*x* − 0.67	0.9998	0.30	1.00
Sy	*y* =20,540.50*x* + 0.98	0.9997	0.29	0.97
Cm	*y* =32,964.76*x* − 2.39	0.9992	0.52	1.57

^1^ LOD-limit of detection; ^2^ LOQ-limit of determination; CA—(+)-Catechin; ECA—Epicatechin; Q-ru—Quercetin-3-rutinoside; Q-glc—Quercetin-glucoside; Q—Quercetin; Km—Kaemferol; L—Luteolin; Caff—Caffeic acid; Sy—Syringic acid; Cm—p-Coumaric acid.

## Data Availability

The original contributions presented in the study are included in the article; further inquiries can be directed to the corresponding author.
